# Supplementary material for: Content-rich biological network constructed by mining PubMed abstracts
Source: BMC Bioinformatics. 2004 Oct 8;5:147. doi: 10.1186/1471-2105-5-147 (PMC528731; doi:10.1186/1471-2105-5-147)
Supplement: Additional File 5 — The original Chilibot query results of the term "long-term potentiation (LTP)" and 22 other terms, limiting the latest references analyzed to the years 1990, 1995, 2000, and 2004. [file 1471-2105-5-147-S5.bz2 › chilibotAdditionalFile5/ltp2000/html/LTP_CREB.html]

 


 **LTP** and **CREB** 
  
Found 30 abstracts in PubMed,  **30 abstracts were retrieved and analyzed**.  


---

 Search Google  |
 PDF files only 
|  EDU domain only 

---

**Interactive relationship** (e.g. stimulation, inhibition, etc)

- We also show that inhibition of the MAPK ERK cascade by the MAPK kinase MEK inhibitor SL327 prevented  **CREB**  and Elk 1 phosphorylation, and  **LTP**  dependent gene induction, resulting in rapidly decaying  **LTP** .  Ref: 10844026 J Neurosci, 2000
- We, and others, have shown that this transcriptional switch is conserved, at least in part, in the regulation of synaptic plasticity in mice  **CREB**  is implicated in activation of genes required for  **LTP** , a model for synaptic plasticity in the mammalian hippocampus.  Ref: 9759326 C R Acad Sci IIIC R Acad Sci III, 2000
- Both pCREB immunocytochemical staining and Western blotting showed that  **CREB**  is rapidly and transiently phosphorylated after induction of long term potentiation  [ **LTP** ] .  Ref: 9497014 J Neurosci Methods, 1997
- CaMKIV dependent activation of  **CREB**  is a key event in the expression of genes involved in the processes of T cell activation and neuronal long term potentiation  [ **LTP** ] .  Ref: 10191497 Biometals, 1998
- These results suggest that NO contributes to L  **LTP**  by stimulating guanylyl cyclase and cGMP dependent protein kinase, which acts in parallel with PKA to increase phosphorylation of the transcription factor  **CREB** .  Ref: 10575022 J Neurosci, 1999
- The gene encoding tissue type plasminogen activator t PA is an immediate response gene, downstream from  **CREB**  1 and other constitutively expressed transcription factors, which is induced in the hippocampus during the late phase of long term potentiation  [ **LTP** ]  L  **LTP** .  Ref: 8710934 Proc Natl Acad Sci U S A, 1996
- Only stimuli that generated nondecremental  **LTP**  promoted a sustained hyperphosphorylation of  **CREB**  but not stimuli that produced decremental  **LTP** .  Ref: 10377374 J Neurosci, 1999

**Parallel relationship** (e.g. studied together, co-existance, homology, etc.)

- Consistent with models claiming a role for long term potentiation  [ **LTP** ]   **LTP**  in memory,  **LTP**  in hippocampal slices from  **CREB**  mutants decayed to baseline 90 min after tetanic stimulation.  Ref: 7923378 Cell, 1994
- Contrary to what was expected, we failed to show any difference in the levels of phosphorylated  **CREB**  after  **LTP**  or following endogenous synaptic facilitation stimulated by novelty.  Ref: 10491575 J Neurosci Res, 1999
- Given the possible role of altered gene expression in the late phase of  **LTP**  and in long term memory, we evaluated the capacity of the MAPK ERK extracellular signal regulated kinase to regulate phosphorylation of the transcription factor cAMP response element binding protein  **CREB**  in hippocampal area CA1.  Ref: 11016081 Acta Neurobiol Exp (Wars), 2000
- Design of a multiple slice interface chamber and application for resolving the temporal pattern of  **CREB**  phosphorylation in hippocampal long term potentiation  [ **LTP** ] .  Ref: 9497014 J Neurosci Methods, 1997
- vgf gene expression is stimulated in vitro by NGF and the ras map kinase signaling cascade through a  **CREB**  dependent mechanism, while in vivo, VGF mRNA levels are regulated by neuronal activity, including long term potentiation  [ **LTP** ] , seizure, and injury.  Ref: 10882540 Front Neuroendocrinol, 2000
- Although high levels of mRNA encoding CREBA133 lead to downregulation of endogenous  **CREB** , expression of  **LTP**  and depotentiation were unaltered in BLA of these transgenic animals.  Ref: 10947828 Eur J Neurosci, 2000
- In this study we investigated whether the activation of  **CREB**  by phosphorylation may have a role in the development of rat perforant path stimulated  **LTP**  and associated dentate granule cell krox 24 mRNA expression.  Ref: 10491575 J Neurosci Res, 1999
- In the absence of the alpha delta  **CREB**  isoforms, performance is impaired in long term memory tasks and the long term maintenance of long term potentiation  [ **LTP** ]   **LTP**  is impaired in the hippocampus.  Ref: 10355905 Cereb CortexCereb Cortex, 1999
- Together, we have resolved the spatial and temporal dynamics of  **CREB**  phosphorylation during hippocampal  **LTP** , showing that the transcription factor  **CREB**  is specifically recruited at two distinct time points in some forms of hippocampal synaptic plasticity in vivo.  Ref: 10377374 J Neurosci, 1999
- Furthermore, the full expression of the late phase of long term potentiation  [ **LTP** ]  L  **LTP**  and L  **LTP**  associated CRE mediated transcription requires ERK activation, suggesting that the activation of  **CREB**  by ERK plays a critical role in the formation of long lasting neuronal plasticity.  Ref: 9808472 Neuron, 1998
- Thus, MAPK ERK activation is required for  **LTP**  dependent transcriptional regulation and we suggest this is regulated by two parallel signaling pathways, the MAPK ERK Elk 1 pathway targeting SRE and the MAPK ERK  **CREB**  pathway targeting CRE.  Ref: 10844026 J Neurosci, 2000
- Nitric oxide signaling contributes to late phase  **LTP**  and  **CREB**  phosphorylation in the hippocampus.  Ref: 10575022 J Neurosci, 1999
- Unlike  **CREB**  alpha delta deficient mice whose main defect is in long term potentiation  [ **LTP** ] , the widespread abnormalities in ATF 2 mutant mice demonstrate its absolute requirement for skeletal and central nervous system development, and for maximal induction of select genes with CRE sites, such as E selectin.  Ref: 8538792 Nature, 1996
- However, despite impaired  **LTP**  and  **CREB**  activation, CaMKIV Gr deficient mice exhibited no obvious deficits in spatial learning and memory.  Ref: 10964952 J Neurosci, 2000
- However, direct evidence for  **CREB**  phosphorylation during hippocampal long term potentiation  [ **LTP** ]   **LTP**  in vivo is sparse.  Ref: 10377374 J Neurosci, 1999
